# Beyond the Kidney and Lung: Cutaneous and Mucosal Clues to Human Hantavirus Disease

**DOI:** 10.1111/ijd.70523

**Published:** 2026-06-09

**Authors:** Giuseppe Gallo, Pietro Quaglino, Simone Ribero

**Affiliations:** ^1^ Dermatology Unit, Department of Medical Sciences University of Turin Turin Italy; ^2^ Department of Medical and Surgical Sciences Alma Mater Studiorum, University of Bologna Bologna Italy

**Keywords:** dermatology, facial flushing, hantavirus, hantavirus pulmonary syndrome, hemorrhagic fever with renal syndrome, mucosal bleeding, orthohantavirus, petechiae, purpura

## Abstract

Hantaviruses are rodent‐borne viruses that cause two overlapping vascular syndromes in humans: hemorrhagic fever with renal syndrome, including nephropathia epidemica, and hantavirus pulmonary/cardiopulmonary syndrome. Although dermatologists are rarely central to hantavirus care, early visible abnormalities may occur on the skin, mucosa, and ocular surface. This narrative review synthesizes the dermatologic and mucocutaneous manifestations of human hantavirus disease, with emphasis on signs that should prompt diagnostic suspicion in dermatology, emergency, travel‐medicine, and infectious‐disease settings. The dermatologic signal is strongest in hemorrhagic fever with renal syndrome, where facial or upper‐torso flushing, conjunctival and pharyngeal injection, petechiae, purpura, ecchymoses, and mucosal bleeding may appear during the febrile or hemorrhagic phases. These findings are not pathognomonic, but their diagnostic value increases when they occur with rodent or outbreak exposure, thrombocytopenia or coagulopathy, and renal abnormalities such as proteinuria, hematuria, or acute kidney injury. In hantavirus pulmonary/cardiopulmonary syndrome, particularly infections caused by New World hantaviruses, visible cutaneous involvement is less prominent and may be absent despite severe pulmonary endothelial disease. We propose a practical dermatology‐oriented trigger for suspicion based on exposure history, abrupt febrile illness, nephro‐pulmonary involvement, thrombocytopenia, and vascular mucocutaneous findings. Hantavirus should be considered when otherwise unexplained vascular skin or mucosal signs occur in a febrile patient with thrombocytopenia, renal involvement, hypoxemia, or compatible exposure. The skin rarely diagnoses hantavirus alone, but it may shorten time to testing, supportive care, and public‐health notification.

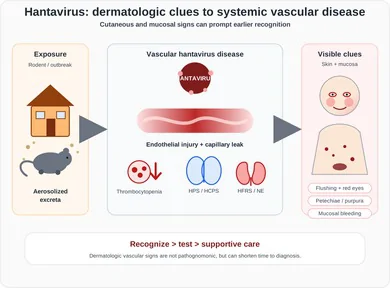

## Capsule Summary

- Hantavirus‐associated skin findings are usually vascular rather than primary exanthematous lesions. Facial flushing, conjunctival and pharyngeal injection, petechiae, purpura, ecchymoses, and mucosal bleeding are the most consistent dermatologic or mucocutaneous clues in hemorrhagic fever with renal syndrome.
- Hantavirus pulmonary/cardiopulmonary syndrome is predominantly a lung‐endothelial disease; the skin may be normal. The absence of rash should not lower suspicion when fever, thrombocytopenia, pulmonary symptoms, and rodent exposure coexist.
- Dermatologists can accelerate diagnosis by linking a febrile hemorrhagic or vasodilatory mucocutaneous pattern with rodent exposure, thrombocytopenia, early proteinuria or hematuria, acute kidney injury, or rapidly evolving hypoxemia.
- Dermatology‐specific evaluation should prioritize morphology, distribution, diascopy, mucosal inspection, and serial documentation; dermoscopy and biopsy may support differential diagnosis but are not substitutes for hantavirus testing and systemic assessment.

## Graphical abstract

Rodent exposure and aerosolized excreta can lead to hantavirus infection with systemic endothelial injury and capillary leak, resulting in thrombocytopenia and syndromes such as HPS/HCPS and HFRS/NE. Early dermatologic and mucosal findings, including flushing, conjunctival injection, petechiae/purpura, and mucosal bleeding, may provide important clues for prompt recognition, testing, and supportive care.

## 1 Introduction

Viral hemorrhagic fevers are often read first on the skin. In dermatology, however, hantavirus disease has remained a peripheral topic, usually mentioned alongside tropical viral infections rather than examined as a dermatologic diagnostic problem in its own right. A classic review in the Journal of the American Academy of Dermatology placed the hantavirus complex among Bunyaviridae infections with mucocutaneous relevance and noted that hantavirus hemorrhagic fever with renal syndrome (HFRS) may present with facial flushing, a petechial rash often limited to the axilla, purpura, ecchymoses, and mucosal bleeding, whereas the skin is largely unaffected in hantavirus pulmonary syndrome (HPS) [1]. That succinct observation still captures the central paradox: hantavirus is a vascular infection, but its cutaneous literature is scattered and thin.

Hantaviruses are globally distributed, negative‐sense RNA viruses maintained in chronically infected reservoir hosts, especially rodents, with human infection typically following inhalation of aerosolized excreta. The clinical spectrum is traditionally divided into HFRS, seen mainly in Eurasia, and HPS/HCPS, seen mainly in the Americas; this distinction is clinically useful but imperfect because endothelial dysfunction, thrombocytopenia, capillary leakage, and organ‐specific vascular injury underlie both syndromes [2, 3, 4, 5]. Current taxonomy places medically important rodent‐borne hantaviruses in the family Hantaviridae and genus *Orthohantavirus*, but the term hantavirus remains entrenched in clinical practice.

Renewed public attention followed a 2026 multi‐country cluster of Andes virus (ANDV) infection linked to cruise‐ship travel. The World Health Organization reported eight cases, including six laboratory‐confirmed infections and three deaths, as of May 8, 2026; the European Centre for Disease Prevention and Control (ECDC) subsequently updated the situation to 12 reported cases, including 10 confirmed and two probable cases, with three deaths, as of May 24, 2026 [6, 7]. ECDC also noted that additional cases may still be identified after passengers and crew returned home because of the long ANDV incubation period and the possibility of onboard exposure, while the risk to the European Union (EU)/European Economic Area (EEA) general population remained very low [7]. For dermatologists, the question is not whether hantavirus produces a disease‐specific eruption; it usually does not. The more relevant question is whether visible vascular, hemorrhagic, or mucosal signs can help clinicians suspect the diagnosis before renal or pulmonary deterioration becomes irreversible.

Historically, HFRS entered modern medicine through the Korean War and the subsequent isolation of Hantaan virus; HPS was recognized rapidly after the 1993 Four Corners outbreak, when a previously unidentified hantavirus was linked to a fulminant pulmonary syndrome [8, 9, 10]. These discoveries framed hantavirus as a nephrologic and critical‐care entity. The present article reframes the infection for dermatology by synthesizing reported cutaneous and mucosal manifestations, explaining their vascular mechanisms, and proposing a practical bedside trigger for suspicion.

## 2 Methods

This article was designed as a dermatology‐centered scoping review and clinical synthesis. We searched PubMed/MEDLINE, Google Scholar, journal websites, and official public‐health resources from inception through May 2026. Search terms included hantavirus, orthohantavirus, hemorrhagic fever with renal syndrome, nephropathia epidemica, hantavirus pulmonary syndrome, hantavirus cardiopulmonary syndrome, Puumala, Hantaan, Dobrava, Seoul, Sin Nombre, Andes, and combinations with skin, cutaneous, dermatologic, rash, exanthem, petechiae, purpura, ecchymosis, flushing, conjunctival, ocular, mucosal, bleeding, hemorrhage, thrombocytopenia, endothelial, vascular permeability, dermoscopy, biopsy, histopathology, differential diagnosis, children, elderly, and immunocompromised.

We included human clinical studies, case series, reviews, mechanistic studies, outbreak investigations, and public‐health documents when they contained information relevant to dermatologic morphology, mucosal or ocular‐surface findings, hemorrhagic manifestations, timing, pathophysiology, differential diagnosis, or clinical actionability. Animal‐only studies were considered only when they clarified mechanisms directly relevant to human vascular leakage. Because dermatologic terminology was heterogeneous, cutaneous findings were grouped into clinically interpretable patterns rather than pooled quantitatively.

The extracted domains were hantavirus syndrome, virus or geographic context when available, morphology, anatomic distribution, timing in the clinical course, associated laboratory abnormalities, organ involvement, severity implications, and diagnostic caveats. We additionally extracted information relevant to dermatology‐specific diagnostic modalities, pediatric and older patients, immunocompromised hosts, and practical dermatologic differential diagnosis. No formal meta‐analysis was attempted, because the literature rarely used skin findings as a primary endpoint and because definitions of rash, petechiae, flushing, and mucosal bleeding varied across studies.

## 3 Results

### 3.1 Clinical Syndromes and the Dermatologic Problem

HFRS spans severe disease due to Hantaan or Dobrava viruses, moderate disease due to Seoul virus, and the often milder but still clinically significant Puumala virus syndrome known as nephropathia epidemica. HPS/HCPS, caused by New World hantaviruses such as Sin Nombre and Andes viruses, is dominated by noncardiogenic pulmonary edema, shock, and hypoxemia. Dermatologic signs are therefore expected to differ by syndrome: HFRS has a more visible hemorrhagic and renal‐vascular phenotype, whereas HPS/HCPS may remain dermatologically silent until systemic collapse.

Large clinical cohorts and reviews of Puumala virus infection consistently emphasize thrombocytopenia, acute kidney injury, and capillary leakage rather than a primary rash. In a cross‐sectional prospective survey of 456 adults with serologically confirmed nephropathia epidemica, acute kidney injury and thrombocytopenia were frequent at presentation, and diagnostic procedures were often undertaken outside nephrology because the early syndrome mimicked other inflammatory or infectious diseases [11]. Contemporary reviews of Puumala‐associated coagulopathy describe low platelet count, altered coagulation, endothelial activation, and acute kidney injury; bleeding is usually mild but clinically relevant [12]. Experimental and translational platelet studies further support the concept that thrombocytopenia and platelet dysfunction are central to the visible hemorrhagic phenotype [13, 14].

### 3.2 Facial Flushing, Conjunctival Injection, and Pharyngeal Injection

The earliest dermatologic clue in HFRS is often not a classic exanthem but a vasodilatory complex: facial or cervicothoracic flushing, conjunctival injection, and pharyngeal injection (Table 1). In clinical dermatology, this pattern should be interpreted as a blanchable vascular erythema arising during the febrile or toxic phase. It may be accompanied by headache, retro‐orbital discomfort, blurred vision, sore throat, abdominal pain, back pain, nausea, vomiting, and rapidly evolving thrombocytopenia.

**TABLE 1:** Dermatologic and mucocutaneous manifestations of human hantavirus disease.

Manifestation	Typical clinical description	Diagnostic value	Important caveats
Facial or upper‐torso flushing	Blanchable erythema during early febrile/toxic phase; may accompany severe headache, back pain, abdominal pain, and nausea	Early visible clue to HFRS when paired with conjunctival/pharyngeal injection, thrombocytopenia, proteinuria, hematuria, or rodent exposure	Nonspecific; overlaps with dengue, drug eruption, toxic shock, sepsis, measles, and other viral exanthems
Conjunctival and pharyngeal injection	Red eyes, pharyngeal erythema, ocular discomfort, photophobia, or blurred vision; in Puumala infection, ocular findings may include eyelid edema, chemosis, and transient myopic shift	Helps distinguish a vascular febrile syndrome from a simple exanthem; should prompt urinalysis and platelet count in endemic or exposure‐compatible settings	May be mistaken for viral conjunctivitis, allergy, leptospirosis, adenovirus, or influenza‐like illness
Petechiae	Small nonblanching macules on skin or mucosa; may be axillary, flexural, palatal, gingival, or conjunctival	Most recognizable cutaneous hemorrhagic sign of HFRS/NE; supports testing when fever, thrombocytopenia, and renal findings coexist	Not pathognomonic; dengue, rickettsioses, meningococcemia, leukemia, immune thrombocytopenia, and drug reactions remain key differentials
Purpura and ecchymoses	Larger nonblanching hemorrhagic lesions; usually less common than petechiae	Suggests more advanced thrombocytopenia, coagulopathy, or vascular leakage; should escalate systemic evaluation	Palpable purpura or necrosis should broaden evaluation toward vasculitis, thrombotic microangiopathy, meningococcemia, or purpura fulminans
Mucosal bleeding	Epistaxis, gingival bleeding, subconjunctival hemorrhage, metrorrhagia, hematuria, melena, or hematemesis	Connects dermatologic observation to systemic hemostasis; hematuria and proteinuria strongly support renal‐vascular involvement	Severe bleeding is not universal, especially in Puumala infection; absence of bleeding does not exclude hantavirus
Normal skin in HPS/HCPS	No rash or only subtle vascular findings despite severe prodrome, thrombocytopenia, hemoconcentration, dyspnea, and hypoxemia	A negative dermatologic examination should not delay testing when pulmonary syndrome and rodent/outbreak exposure are present	A prominent morbilliform, vesicular, or necrotic eruption argues for alternative or additional diagnoses

Abbreviations: HCPS, hantavirus cardiopulmonary syndrome; HFRS, hemorrhagic fever with renal syndrome; HPS, hantavirus pulmonary syndrome; NE, nephropathia epidemica.

This finding is diagnostically useful because it precedes or coincides with the period when renal markers begin to declare themselves. A patient with abrupt fever, severe headache or back pain, facial erythema, red eyes, pharyngeal injection, thrombocytopenia, proteinuria, or hematuria deserves targeted questioning about rodent exposure, cleaning of sheds or cabins, occupational exposure, travel, camping, farming, forestry, military activity, and outbreak contacts. In isolation, flushing overlaps with dengue, measles, toxic shock, drug eruption, viral exanthems, leptospirosis, rickettsioses, and sepsis. In the correct exposure context, it becomes one of the few visible early clues to HFRS [1, 4, 5].

### 3.3 Petechiae, Purpura, and Ecchymoses

Petechiae are the most recognizable cutaneous manifestation of hantavirus‐associated hemorrhagic disease (Table 1). Reports describe petechiae on skin and mucosa, sometimes with an axillary or flexural accent, and sometimes with palatal, gingival, or conjunctival involvement. Purpura and ecchymoses occur less consistently and should raise concern for more advanced thrombocytopenia, coagulopathy, vascular leakage, or concomitant disseminated intravascular coagulation.

In nephropathia epidemica, petechiae of the skin and mucosa, ecchymoses, conjunctival bleeding, and epistaxis have been described in approximately one‐third of patients in reviews of Finnish experience, although bleeding is usually mild compared with classic severe HFRS [12]. Importantly, the morphology may be subtle. A few nonblanching macules in the axillae, a palatal petechial cluster, or unexplained subconjunctival hemorrhage can be more informative than a widespread rash if they appear in a febrile patient with thrombocytopenia and renal abnormalities.

Dermatologists should resist the temptation to classify these lesions simply as viral exanthem. The more accurate description is microvascular bleeding in a systemic capillary‐leak syndrome. That distinction matters because it immediately changes the work‐up: complete blood count with platelets, creatinine, urinalysis for proteinuria and hematuria, liver enzymes, coagulation studies, oxygen saturation, and exposure history should be obtained before skin biopsy is considered.

### 3.4 Mucosal Bleeding and Ocular‐Surface Findings

Mucosal hemorrhage is a high‐yield sign because it links the skin examination to systemic hemostasis (Table 1). Epistaxis, gingival bleeding, conjunctival hemorrhage, metrorrhagia, hematuria, melena, and hematemesis have all been reported in hantavirus‐associated HFRS/NE, with severity varying by virus, host factors, and clinical phase [12]. Hematuria deserves special attention: while not a dermatologic lesion, it is the mucosal‐renal counterpart of petechiae and can help anchor a suspected HFRS diagnosis when cutaneous findings are faint.

Ocular involvement is also relevant to dermatologists because periocular edema, conjunctival injection, chemosis, and subconjunctival hemorrhage are visible at the bedside. In Puumala virus infection, ophthalmic studies have documented blurred vision, transient myopic shift, eyelid edema, conjunctival injection, chemosis, subconjunctival bleeding, and anterior uveitis [15, 16]. A febrile patient with red eyes, acute visual blurring, thrombocytopenia, and renal findings should therefore not be reflexively attributed to nonspecific viral conjunctivitis, particularly in endemic regions.

### 3.5 HPS/HCPS: The Negative Dermatologic Finding Is Itself Informative

In HPS/HCPS, dermatologic manifestations are less reliable. The classic clinical trajectory is a prodrome of fever, myalgia, headache, and gastrointestinal symptoms followed by abrupt pulmonary edema and shock. Cutaneous hemorrhage is not a defining feature of the syndrome, and the skin may appear normal. This absence has diagnostic value: a patient with fever, thrombocytopenia, hemoconcentration, rodent exposure, and rapidly progressive dyspnea should be tested for hantavirus even if there is no rash (Table 1).

When conjunctival injection, petechiae, or mucosal bleeding are present in suspected HPS/HCPS, they should be considered supportive but not required signs. Conversely, a prominent morbilliform or vesiculopustular eruption should widen the differential diagnosis toward dengue, chikungunya, measles, enteroviruses, rickettsioses, meningococcemia, leptospirosis, viral hemorrhagic fevers other than hantavirus, drug reactions, or sepsis‐associated purpura.

### 3.6 Mechanistic Bridge: Why a Lung‐Kidney Virus Appears on the Skin

The dermatologic manifestations of hantavirus infection are best explained by endothelial dysfunction and platelet biology. Hantaviruses infect endothelial cells nonlytically, and pathogenic viruses use beta3 integrin pathways that are relevant to endothelial cells and platelets [17]. Experimental work has shown that pathogenic Hantaan, Andes, and New York‐1 viruses can sensitize endothelial cells to vascular endothelial growth factor, increasing permeability, whereas angiopoietin‐1 and sphingosine‐1‐phosphate can counteract this effect in vitro [18]. Infected endothelial cells can also recruit quiescent platelets, and pathogenic Andes and Hantaan viruses induce adherens‐junction disassembly by directing VE‐cadherin internalization [19, 20].

These mechanisms produce the bedside phenotype: blanchable flushing from vascular dysregulation, petechiae and purpura from platelet consumption or dysfunction plus endothelial leakage, mucosal bleeding from fragile microvasculature, and pulmonary or renal edema from organ‐specific capillary leak. The same mechanistic frame also explains why the skin is an imperfect marker: the organ most affected by vascular leakage differs by virus, host response, and endothelial bed [21].

### 3.7 Andes Virus and Transmission Context

Andes virus requires separate mention because it has been associated with person‐to‐person transmission under close‐contact conditions, unlike most other human hantaviruses. A 2020 investigation of an Argentine outbreak reported 34 infections and 11 deaths with reconstructed person‐to‐person transmission chains [22]. A later systematic review concluded that the overall evidence for human‐to‐human transmission is limited, specific to Andes virus, and vulnerable to confounding by shared rodent exposure, but that precautionary infection‐control measures may be prudent in suspected cases [23].

For dermatologists, this means that the exposure history should not stop at rodents. In the setting of a recognized Andes virus cluster, recent close contact with a symptomatic patient, participation in shared travel, or exposure to a confined outbreak environment may become relevant. Nevertheless, the core diagnostic pattern remains clinical: fever, thrombocytopenia, vascular leakage, and pulmonary or renal involvement, with or without visible mucocutaneous signs.

### 3.8 A Dermatology Diagnostic Framework: The HANTA‐DERM Trigger

Because no single lesion is pathognomonic, suspicion should be pattern‐based. We propose the HANTA‐DERM trigger as a pragmatic bedside framework rather than a formal diagnostic score (Table 2). Hantavirus testing or urgent infectious‐disease consultation should be considered when a patient has compatible systemic illness plus at least one meaningful exposure and at least one vascular dermatologic or mucosal clue.

**TABLE 2:** Hanta‐derm trigger for dermatology‐oriented suspicion of hantavirus disease.

Trigger element	What to ask or examine	Actionable interpretation
H—History of exposure	Rodent droppings or urine; cleaning sheds, barns, cabins, garages, basements, or ship compartments; camping; farming; forestry; military activity; endemic travel; recognized outbreak; close contact in an Andes virus cluster	A compatible exposure makes otherwise nonspecific vascular skin signs clinically meaningful
A—Abrupt febrile illness	Fever, chills, severe headache, myalgia, back or abdominal pain, nausea, vomiting, diarrhea, or visual symptoms	The early phase can mimic influenza, dengue, leptospirosis, sepsis, or drug eruption.
N—Nephro‐pulmonary involvement	Proteinuria, hematuria, rising creatinine, oliguria, dyspnea, cough, hypoxemia, pulmonary infiltrates, or shock	Renal findings favor HFRS/NE; rapid pulmonary edema favors HPS/HCPS. Overlap can occur
T—Thrombocytopenia/coagulopathy	Low platelets, hemoconcentration, prolonged coagulation times, elevated D‐dimer, or clinical bleeding	Laboratory vascular injury should transform a rash evaluation into an urgent systemic work‐up
A—Axillary, palatal, ocular, or mucosal hemorrhage	Petechiae in axillae or flexures; palatal petechiae; gingival bleeding; subconjunctival hemorrhage; epistaxis; hematuria	Small localized lesions may be more diagnostic than a diffuse nonspecific rash
DERM—Dermatology action	Document morphology and distribution; avoid unnecessary biopsy; order CBC, creatinine, urinalysis, liver enzymes, coagulation studies, and oxygen saturation; discuss hantavirus testing and notification	Dermatology adds value by shortening time to recognition, testing, supportive care, and public‐health response

The highest‐yield dermatologic constellation is: abrupt fever; facial or upper‐torso flushing; conjunctival or pharyngeal injection; petechiae, purpura, ecchymoses, or mucosal bleeding; thrombocytopenia; and either proteinuria, hematuria, rising creatinine, or early hypoxemia. This pattern is particularly persuasive in a patient from an endemic area, after cleaning rodent‐contaminated spaces, after camping or agricultural exposure, or during an outbreak investigation.

Clinicians evaluating suspected HPS/HCPS should contact public‐health authorities when compatible symptoms and rodent or outbreak exposure are present; diagnostic testing typically relies on hantavirus‐specific serology and, in selected early or outbreak cases, molecular or immunohistochemical methods. Current clinical guidance emphasizes early recognition, supportive care, intensive monitoring for HPS/HCPS, and early care for HFRS because no dermatologic procedure can substitute for timely systemic management [24, 25].

### 3.9 Dermatology‐Specific Diagnostic Modalities

At the bedside, the most useful dermatology‐specific tools are descriptive rather than confirmatory. Diascopy can separate blanchable flushing or vascular erythema from nonblanching petechiae and purpura. Dermoscopy may help document punctate red‐purple macules, red dots or globules, hemorrhagic crust, and purpuric structureless areas, but no dermoscopic pattern is specific for hantavirus. Oral mucoscopy, careful inspection of the palate and gingivae, conjunctival examination, and serial clinical photography can improve documentation of subtle mucocutaneous findings and lesion evolution. When visual blurring, chemosis, uveitis, or subconjunctival hemorrhage is present, ophthalmologic assessment remains appropriate because ocular involvement has been described in Puumala virus infection [15, 16].

Histopathology is not a first‐line diagnostic test for suspected hantavirus. If biopsy is performed because lesions are palpable, necrotic, vesicular, ulcerated, persistent, or discordant with the systemic picture, the expected contribution is exclusion of mimics rather than confirmation of hantavirus. Possible findings would be nonspecific microvascular injury, including erythrocyte extravasation, edema, endothelial swelling, sparse perivascular inflammation, and variable fibrin thrombi; frank leukocytoclastic vasculitis should prompt evaluation for immune‐complex vasculitis, rickettsioses, meningococcemia, drug reaction, or thrombotic microangiopathy. Direct immunofluorescence may help exclude immune‐complex disease. Tissue immunohistochemistry or molecular testing belongs to reference laboratory or pathology workflows and should not delay serology, polymerase chain reaction (PCR) when available, platelet count, renal and pulmonary assessment, and public‐health notification [24, 25, 26].

### 3.10 Pediatric, Immunocompromised, and Older Patients

Pediatric presentations are uncommon and dermatologic descriptions are sparse. In children, HFRS/nephropathia epidemica may be milder than in adults, and the initial complaint may be fever, abdominal pain, vomiting, headache, or malaise rather than a clearly hemorrhagic syndrome. Subtle palatal petechiae, conjunctival injection, or a few nonblanching macules should therefore be interpreted with platelet count, urinalysis, renal markers, and family‐level exposure history because children may not report rodent contact or high‐risk cleaning activities. In suspected HPS/HCPS, a normal skin examination should not reassure clinicians when fever, thrombocytopenia, gastrointestinal symptoms, dyspnea, and compatible exposure coexist [5, 24, 25].

In immunocompromised patients, the evidence base is limited. Dermatologists should anticipate diagnostic noise: drug eruptions, opportunistic infections, malignancy‐associated thrombocytopenia, transplant‐related thrombotic microangiopathy, anticoagulation, and impaired inflammatory responses can all obscure or mimic hantavirus‐associated vascular lesions. A lower threshold for biopsy may be reasonable when lesions are atypical or persistent, but biopsy should be coordinated with platelet/coagulation status, infection‐control procedures, and systemic diagnostic testing rather than used as a screening test.

Older adults deserve separate attention because comorbidity, antiplatelet or anticoagulant therapy, frailty, and baseline actinic or senile purpura can make vascular lesions easy to dismiss. Conversely, mild petechiae or mucosal bleeding in an older febrile patient with thrombocytopenia and renal or pulmonary involvement should not be attributed automatically to age‐related purpura or medication effect. In the 2026 cruise‐ship cluster, WHO emphasized that older age and comorbidities may increase the risk of severe disease in this setting [6].

### 3.11 Literature‐Based Clinical Scenarios

Because this review does not report a primary institutional case series, the following scenarios are literature‐based clinical vignettes rather than new patient observations. They are included to make the diagnostic framework clinically usable while avoiding the implication that unpublished cases or identifiable patient photographs are being presented.

Scenario 1: HFRS/nephropathia epidemica. An adult from an endemic European region presents with abrupt fever, severe headache or back pain, facial flushing, red eyes, scattered axillary petechiae, palatal petechiae, thrombocytopenia, proteinuria, and microscopic hematuria after cleaning a rodent‐contaminated shed. The dermatologic contribution is not a pathognomonic rash but recognition that flushing plus nonblanching mucocutaneous lesions in a febrile thrombocytopenic patient should trigger urinalysis, renal testing, exposure history, and hantavirus serology.

Scenario 2: HPS/HCPS or Andes virus exposure. A traveler or cruise passenger with fever, myalgia, gastrointestinal symptoms, thrombocytopenia, and early hypoxemia has no rash. The negative skin examination should not delay hantavirus testing or escalation of care because New World HPS/HCPS may be dermatologically silent while pulmonary capillary leakage is evolving.

Scenario 3: mimic requiring a broader dermatologic differential. A febrile patient with extensive palpable purpura, necrosis, eschar, vesicles, or a morbilliform eruption without renal‐pulmonary features or rodent/outbreak exposure should prompt broader evaluation for dengue, rickettsioses, leptospirosis, meningococcemia, other viral hemorrhagic fevers, drug reaction, vasculitis, hematologic disease, or sepsis‐associated coagulopathy (Table 3).

**TABLE 3:** Dermatology‐oriented differential diagnosis of hantavirus‐like mucocutaneous presentations.

Differential diagnosis	Dermatologic overlap with hantavirus	Clues favoring the alternative diagnosis	Dermatology‐oriented action
Dengue or severe dengue	Facial flushing, petechiae, purpura, ecchymoses, mucosal bleeding	Mosquito exposure, travel to endemic area, retro‐orbital pain, marked arthralgia, leukopenia, plasma leakage; renal injury usually secondary to severe systemic disease	Ask vector/travel history; obtain platelet count, hematocrit trend, liver enzymes, dengue NS1/serology/PCR; avoid anchoring on hantavirus
Leptospirosis	Conjunctival suffusion, petechiae, purpura, hemorrhage, renal involvement	Freshwater, animal urine, flooding, occupational exposure, calf tenderness, jaundice, aseptic meningitis, Weil disease	Request exposure history; test with serology/PCR as locally available; prioritize antibiotic therapy and renal‐hepatic assessment
Rickettsioses	Fever with petechial, purpuric, or maculopapular eruption	Tick exposure, eschar, acral or palm/sole involvement, centripetal spread, vasculitic necrosis	Inspect scalp, folds, palms/soles, and eschar sites; early empiric doxycycline may be lifesaving
Meningococcemia, purpura fulminans, or bacterial sepsis/DIC	Rapidly progressive nonblanching petechiae, purpura, ecchymoses, mucosal bleeding	Toxic appearance, neck stiffness, hypotension, shock, DIC, retiform purpura, skin necrosis	Treat as emergency; blood cultures and immediate antimicrobials/resuscitation should not wait for dermatologic testing
Other viral hemorrhagic fevers	Petechiae, ecchymoses, mucosal bleeding, conjunctival injection, systemic vascular leak	Travel or residence in endemic areas; tick/livestock exposure; contact with ill persons; high‐consequence public‐health context	Immediate isolation, infection‐control escalation, and public‐health notification according to local guidance
Hematologic or thrombotic disorders	Petechiae, purpura, oral bleeding, ecchymoses.	Blasts, schistocytes, severe isolated thrombocytopenia, hemolysis, neurologic findings, no compatible exposure	CBC with smear, hemolysis panel, coagulation tests, hematology consultation; skin findings are secondary clues.
Drug eruption or severe cutaneous adverse reaction	Morbilliform rash, facial edema, purpura, mucosal involvement	New medication, eosinophilia, liver injury, targetoid lesions, bullae, epidermal detachment, prominent pruritus	Stop culprit drug when appropriate; biopsy if SCAR is suspected; evaluate for DRESS, SJS/TEN, or vasculitic drug reaction
Small‐vessel vasculitis or IgA vasculitis	Palpable purpura, renal involvement, abdominal pain	Dependent palpable purpura, arthralgia, recurrent lesions, immune‐complex pattern, absence of rodent/outbreak exposure	Biopsy a fresh lesion for histology and direct immunofluorescence if platelet/coagulation status permits
Common viral exanthems, enterovirus, measles, COVID‐19/MIS‐C	Fever, conjunctivitis, morbilliform eruption, oral findings	Vaccination status, respiratory outbreak, Koplik spots, hand‐foot‐mouth lesions, lymphadenopathy, marked inflammatory syndrome	Use appropriate isolation and virologic testing; reassess for thrombocytopenia, renal/pulmonary findings, and exposure history before considering hantavirus

## 4 Discussion

**FIGURE 1:**
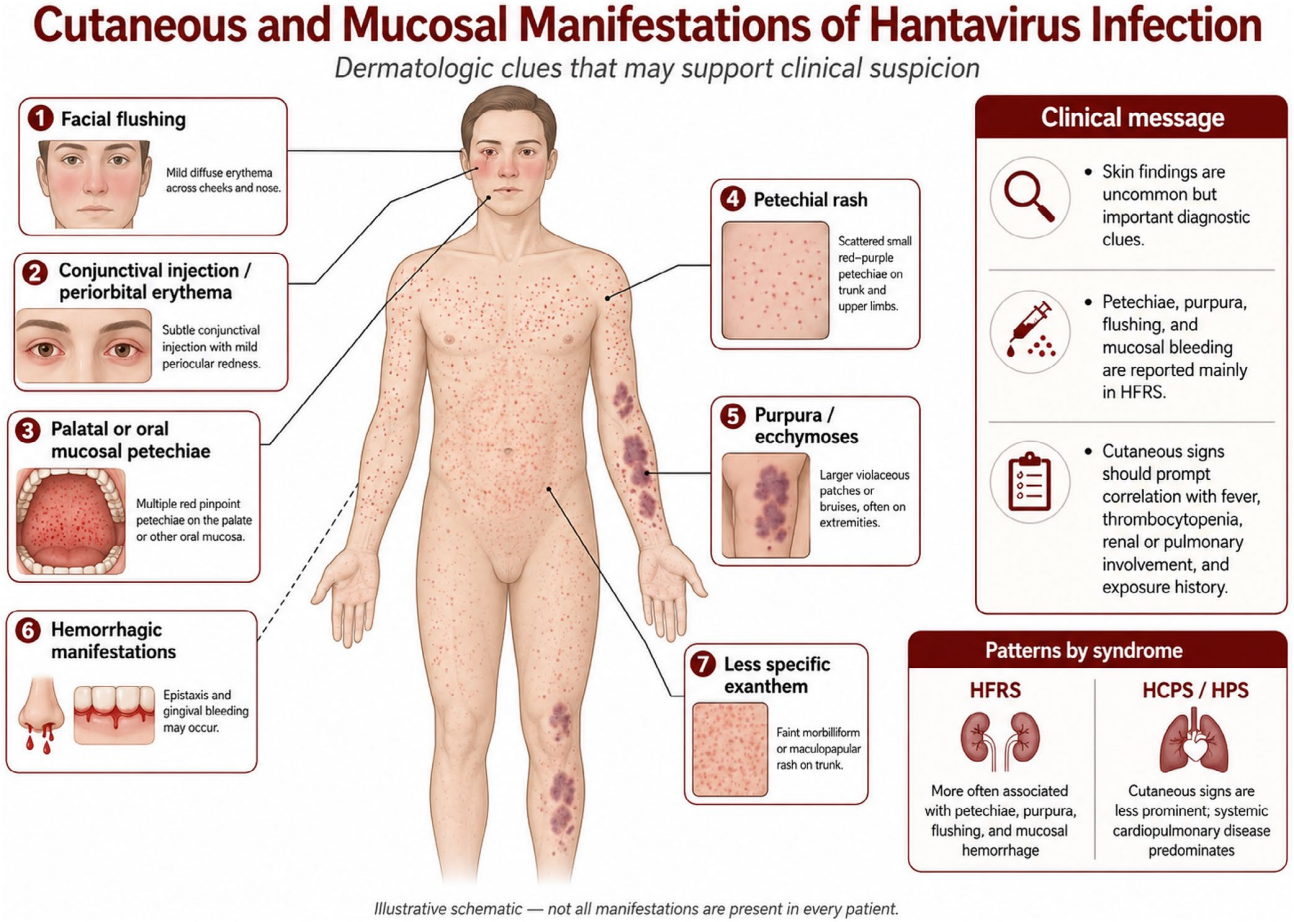
Cutaneous and mucosal manifestations of hantavirus infection.

The dermatology of hantavirus disease is not a catalogue of distinctive eruptions. It is a pattern‐recognition exercise in systemic vascular medicine. The strongest visible clues are vascular and hemorrhagic: flushing, injected conjunctivae, pharyngeal erythema, petechiae, purpura, ecchymoses, and mucosal bleeding (Figure 1). These findings can be transient, localized, or absent, and their absence should never be used to exclude HPS/HCPS.

The main diagnostic value of the skin examination is triage. A dermatologist, emergency physician, or internist who recognizes petechiae in the axillae, palatal purpura, subconjunctival hemorrhage, or facial flushing in a febrile thrombocytopenic patient can ask the exposure questions that are often missed: Was a cabin, cellar, barn, shed, garage, or ship compartment cleaned? Were rodent droppings, nests, or urine present? Was there camping, farming, forestry, military training, or travel to an endemic area? Was there close contact with a patient in a suspected Andes virus cluster?

A dermatology‐specific differential diagnosis is essential because most visible findings in hantavirus are shared with other vascular, hemorrhagic, infectious, and drug‐related conditions (Table 3). Dengue can produce fever, flushing, petechiae, positive tourniquet testing, and mucosal bleeding; leptospirosis can produce conjunctival suffusion, renal injury, jaundice, and hemorrhage; rickettsial infections, meningococcemia, viral hemorrhagic fevers, sepsis, thrombotic microangiopathy, immune thrombocytopenia, drug‐induced thrombocytopenia, vasculitis, and acute leukemia can all mimic parts of the hantavirus phenotype. Hantavirus becomes plausible when the vascular skin pattern is integrated with thrombocytopenia, renal or pulmonary involvement, and exposure epidemiology.

Skin biopsy is rarely the first diagnostic step. In a patient with suspected hantavirus, biopsy may add bleeding risk and is unlikely to provide rapid actionable information. The more useful dermatology intervention is documentation of morphology and distribution, use of noninvasive bedside tools such as diascopy and dermoscopy when helpful, communication of the syndromic concern, and acceleration of laboratory and public‐health testing. When lesions are atypical, persistent, necrotic, palpable, vesicular, or ulcerative, biopsy may be justified to evaluate competing diagnoses, but it should not delay systemic care.

### 4.1 Clinical Photography and Patient‐Safety Considerations

Clinical photographs of true hantavirus‐associated petechiae, purpura, flushing, or mucosal bleeding would be valuable for dermatologists; however, they could not be included in this review because the article is a synthesis of published literature and public‐health documents, not a prospective case series with institutional consented images. Published hantavirus reports rarely contain dermatology‐quality photographs, probably because the disease is uncommon, cutaneous signs may be transient or subtle, and acute management appropriately prioritizes isolation, resuscitation, laboratory confirmation, and public‐health coordination.

The absence of original images should therefore be interpreted as a transparent limitation rather than an omission of available data. In suspected Andes virus infection, limited human‐to‐human transmission has been documented in close‐contact settings, and recent ECDC guidance emphasizes infection‐prevention and control measures for healthcare encounters. Prospective acquisition of clinical photographs during an acute suspected case would require patient consent, institutional approval, personal protective equipment, and coordination with infection‐control teams. For this reason, the manuscript uses a non‐identifiable schematic figure and literature‐based clinical scenarios instead of patient photographs [22, 23, 26].

### 4.2 Strengths and Limitations

This article has several strengths. It focuses on a neglected dermatologic question, separates HFRS from HPS/HCPS, distinguishes primary exanthem from vascular hemorrhagic signs, integrates ocular and mucosal clues, adds dermatology‐specific diagnostic modalities and differential diagnosis, addresses clinically important populations, and connects bedside morphology with endothelial and platelet mechanisms. The proposed HANTA‐DERM trigger is designed for practical suspicion rather than overdiagnosis.

The main limitation is the evidence base. Dermatologic findings are inconsistently described in hantavirus reports, photographs are uncommon, skin findings are rarely primary endpoints, and most cohorts do not provide sensitivity, specificity, predictive values, or timing by lesion type. The absence of real patient images reflects the nature of this literature review and the scarcity of accessible, consented dermatology‐quality photographs in a rare, potentially high‐consequence infection. The available literature therefore supports a diagnostic suspicion framework, not a validated dermatologic scoring system. Future prospective studies should use standardized lesion vocabulary, lesion distribution maps, serial photography, dermoscopy when feasible, platelet and coagulation phenotyping, and linked renal‐pulmonary outcomes.

## 5 Conclusions

Hantavirus disease should be considered in febrile patients with vascular mucocutaneous signs when petechiae, purpura, mucosal bleeding, conjunctival injection, or facial flushing coexist with thrombocytopenia, renal abnormalities, hypoxemia, or rodent/outbreak exposure.

The dermatologic signal is strongest in HFRS/nephropathia epidemica and is usually vascular rather than primarily exanthematous. In HPS/HCPS, the most important dermatologic lesson may be that severe disease can occur with little or no rash.

Dermatologists can add value by documenting morphology, asking exposure‐focused questions, broadening the differential diagnosis, and accelerating systemic testing, supportive care, and public‐health notification.

## Funding

The authors have nothing to report.

## Conflicts of Interest

The authors declare no conflicts of interest.

## Data Availability

The data that support the findings of this study are available from the corresponding author upon reasonable request.
